# An Ultrafast UPLC–MS/MS Method for Characterizing the In Vitro Metabolic Stability of Acalabrutinib

**DOI:** 10.3390/molecules28207220

**Published:** 2023-10-23

**Authors:** Mohamed W. Attwa, Ahmed H. Bakheit, Ali S. Abdelhameed, Adnan A. Kadi

**Affiliations:** Department of Pharmaceutical Chemistry, College of Pharmacy, King Saud University, P.O. Box 2457, Riyadh 11451, Saudi Arabia; abakheit@ksu.edu.sa (A.H.B.); asaber@ksu.edu.sa (A.S.A.); akadi@ksu.edu.sa (A.A.K.)

**Keywords:** acalabrutinib, intrinsic clearance, in vitro half-life, metabolic stability, P450 metabolic program, UPLC–MS/MS, greenness, DEREK, ADME profile, StarDrop software package

## Abstract

Acalabrutinib, commercially known as Calquence^®^, is a pharmacological molecule that has robust inhibitory activity against Bruton tyrosine kinase. The medicine in question was carefully developed by the esteemed pharmaceutical company AstraZeneca. The FDA granted authorization on 21 November 2019 for the utilization of acalabrutinib (ACB) in the treatment of small lymphocytic lymphoma (SLL) or chronic lymphocytic leukemia (CLL) in adult patients. The aim of this study was to develop a UPLC–MS/MS method that is effective, accurate, environmentally sustainable, and has a high degree of sensitivity. The methodology was specifically developed with the intention of quantifying ACB in human liver microsomes (HLMs). The methodology described above was subsequently utilized to assess the metabolic stability of ACB in HLMs in an in vitro environment. The validation procedures for the UPLC–MS/MS method in the HLMs were conducted in accordance with the bioanalytical method validation criteria established by the U.S.- DA. The utilization of the StarDrop software (version 6.6), which integrates the P450 metabolic module and DEREK software (KB 2018 1.1), was employed for the purpose of evaluating the metabolic stability and identifying potential hazardous alarms associated with the chemical structure of ACB. The calibration curve, as established by the ACB, demonstrated a linear correlation across the concentration range of 1 to 3000 ng/mL in the matrix of HLMs. The present study conducted an assessment of the accuracy and precision of the UPLC–MS/MS method in quantifying inter-day and intra-day fluctuations. The inter-day accuracy demonstrated a spectrum of values ranging from −1.00% to 8.36%, whilst the intra-day accuracy presented a range of values spanning from −2.87% to 4.11%. The t_1/2_ and intrinsic clearance (Cl_int_) of ACB were determined through in vitro testing to be 20.45 min and 39.65 mL/min/kg, respectively. The analysis concluded that the extraction ratio of ACB demonstrated a moderate level, thus supporting the recommended dosage of ACB (100 mg) to be administered twice daily for the therapeutic treatment of persons suffering from B-cell malignancies. Several computational tools have suggested that introducing minor structural alterations to the butynoyl group, particularly the alpha, beta-unsaturated amide moiety, or substituting this group during the drug design procedure, could potentially enhance the metabolic stability and safety properties of novel derivatives in comparison to ACB.

## 1. Introduction

**Figure 1:**
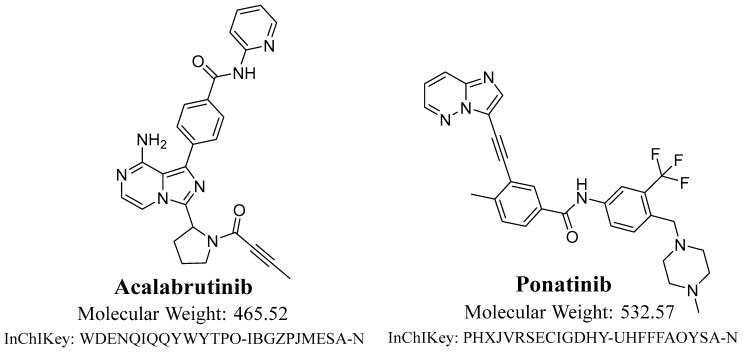
Chemical structure of acalabrutinib and ponatinib (internal standard; IS).

B-cell lymphomas are the most frequent hematologic malignancies. The pharmaceutical therapy of B-cell lymphomas has changed significantly since the development of targeted drugs such as Bruton tyrosine kinase (BTK) inhibitors [1]. BTK signaling is crucial for B-cell proliferation, differentiation, and survival, and it is also essential for leukemic cell survival [2]. Thus, BTK inhibition is a developed therapeutic pathway for the treatment of B-cell malignancies. On 21 November 2019, acalabrutinib (Calquence, AstraZeneca, Cambridge, UK) was approved by the FDA for adults with small lymphocytic lymphoma (SLL) or chronic lymphocytic leukemia (CLL). The regiment of Calquence is that it is administered orally twice a day [3]. Acalabrutinib (ACB; Figure 1) is utilized in the treatment of numerous diseases, comprising mantle cell lymphoma and refractory/relapsed chronic lymphocytic leukemia (small lymphocytic lymphoma) [4,5,6]. The development of ACB aimed to enhance the effectiveness and specificity of the first BTK inhibitors, like ibrutinib [7]. ACB has a narrower spectrum of kinase inhibition compared to ibrutinib. It forms a covalent bond with the cystine 481 residue on the binding site of BTK [8,9].

Although ACB is considered a relatively safe drug in comparison with other anticancer agents, it is associated with numerous adverse effects. The most common and serious adverse effects caused by ACB include neutropenia (23%), serious infections (19%), major hemorrhage (3%), anemia (8%), cardiac arrhythmias (4%), thrombocytopenia (7%), and lymphopenia (7%) [8]. Upon completing an extensive review of the available academic literature, it has been determined that there is a notable absence of published research specifically addressing the estimation of ACB in human liver microsomes (HLMs) in relation to the evaluation of ACB’s metabolic stability. Therefore, it is imperative to develop a methodology that utilizes ultraperformance liquid chromatography–tandem mass spectrometry (UPLC–MS/MS) with the dual objectives of achieving quick analysis and attaining a heightened level of sensitivity. The utilization of this methodology is crucial for accurately measuring the concentration of ACB in a wide range of matrices. The primary aim of the present study was to establish a rapid UPLC–MS/MS method that demonstrates high sensitivity (with a limit of detection as low as 1 ng/mL in a matrix of HLMs) and specificity for assessing the metabolic stability of ACB in a matrix of HLMs. Moreover, the results were validated using in vitro investigations, namely, metabolic incubations, along with in silico analysis employing StarDrop software (version 6.6) developed by Optibrium Ltd. (Cambridge, MA, USA). In contemporary times, there has been an increasing emphasis on the subject of green analytical chemistry (GAC), which aims to address the presence of harmful chemicals, decrease energy usage, and limit the production of waste by employing various analytical techniques [10,11]. The research integrates multiple approaches, specifically the Analytical Eco-Scale (AES), National Environmental Methods Index (NEMI), Red–Green–Blue (RGB), Green Analytical Procedures Index (GAPI), and Analytical Greenness Metric Approach (AGREE) [10]. The AES, GAPI, NEMI, and RGB methodologies displayed a dependence on specific GAC values, as evidenced. The “AGREE” methodology was utilized to assess the degree of environmental sustainability through the evaluation of twelve Greenness Assessment Criteria (GAC) and the assignment of corresponding scores. The UPLC–MS/MS system showcased its environmental sustainability with the implementation of a decreased flow rate of 0.3 mL/min and an impressively short run time of 1 min. The linear relationship between the observed connection in this system was established by conducting experiments across a concentration range of 1 ng/mL to 3000 ng/mL.

In this present study, we investigated the in vitro metabolic stability of ACB utilizing a UPLC–MS/MS methodology. Additionally, we supplemented our findings with data obtained from the DEREK ((KB 2018 1.1) and P450 software (version 6.6) tools. The StarDrop software program, which incorporates the P450 metabolic module and DEREK software, was employed to assess the metabolic stability and identify potential hazardous alarms within the chemical structure of ACB. The assessment of in silico metabolic stability was conducted utilizing the StarDrop software package (version 6.6), with particular emphasis on the in silico P450 program. Metabolic lability refers to the quantification of the efficiency of the product generation phase within the catalytic cycle of CYP3A4. The presence of metabolic lability can be determined by assessing the composite site lability (CSL) value. A decreased CSL value indicates a higher probability of enhanced stability. The aforementioned value was employed as initial evidence to support the significance of conducting practical procedures, such as analytical method development and in vitro incubation with HLMs, in order to optimize resource allocation and time management [12].

The Cl_int_ and in vitro t_1/2_ of ACB were assessed in the present investigation using the UPLC–MS/MS approach [13]. Three distinct models—dispersion, parallel tube, and venous equilibrium—were then used to estimate the in vivo metabolic rate using these parameters [14,15]. An in vitro method (the well-stirred model) was utilized to determine the in vitro t_1/2_ and Cl_int_ of ACB [14,15]. Because of its intrinsic simplicity, this specific model is frequently used in studies pertaining to drug metabolism. According to earlier research [16,17,18,19], the ACB showed a moderate metabolism rate, which led to a favorable in vivo bioavailability and a moderate duration of action. The small extraction ratio of ACB was discovered to be in line with the frequently advised regular dosage of ACB (100 mg), which should be given twice daily to patients diagnosed with B-cell malignancies [20]. The suggested theory for in silico metabolic stability, which matched the outcomes of the in vitro metabolic stability, was supported by screening for structural alarms inside the ACB chemical structure using DEREK software. The absorption, distribution, metabolism, and excretion (ADME) profile of ACB was ascertained by a computer study, which aimed to predict its drug-like characteristics.

## 2. Results and Discussions

### 2.1. In Silico Assessment of ACB Metabolic Lability

**Figure 2:**
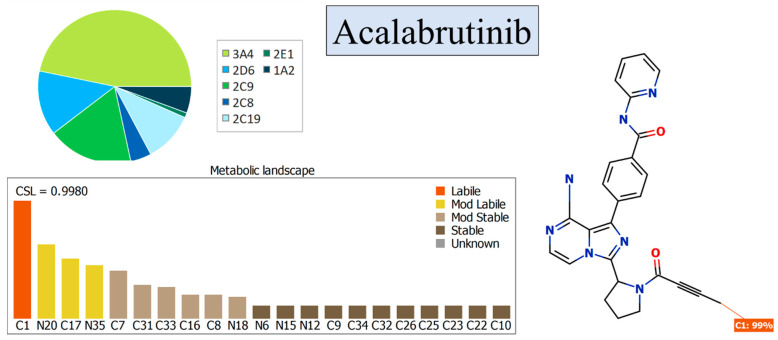
CSL value (0.9980) indicating the high lability of ACB to metabolism. The data were evaluated by the P450 module of the StarDrop software (Cambridge, MA, USA).

The ACB metabolic landscape was utilized to predict the metabolic instability of the active atoms within the ACB chemical structure with respect to CYP3A4 enzymes, as illustrated in the pie chart [21,22,23]. The metabolic stability of ACB was assessed by employing the CSL value of 0.9980, as illustrated in Figure 2. The UPLC–MS/MS approach was employed to assess the metabolic stability of ACB following incubation with the active HLMs matrix. The primary factor influencing the metabolic lability of ACB is the notable metabolic instability reported at the C1 position of the butynoyl group. This assertion is substantiated by the robust connection observed between the metabolic lability and the CSL value of 0.9980, as illustrated in Figure 2. The aforementioned conclusion aligns with the outcomes obtained from the in vitro metabolic experiments, which will be expounded upon in later parts.

### 2.2. In Silico Toxicity Expectation of ACB Using DEREK Software

**Figure 3:**
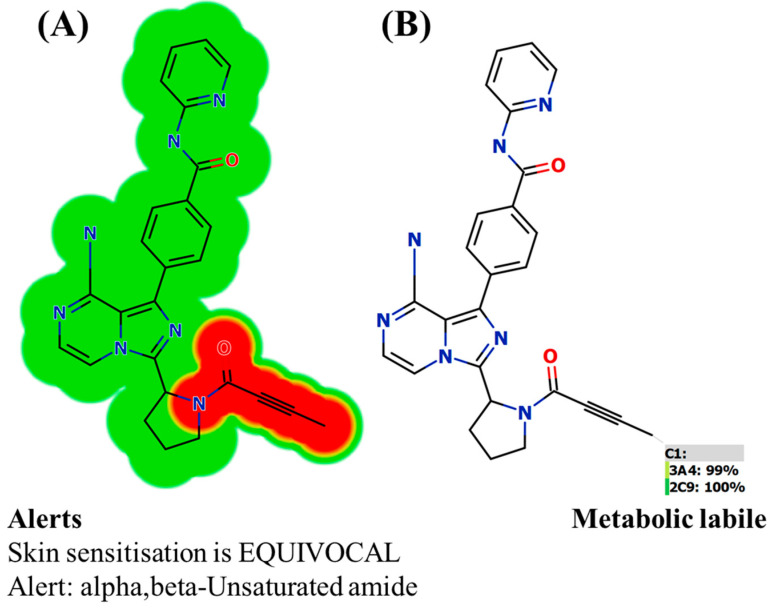
Structural alerts of ACB using DEREK software highlighted in red color (**A**). P450 lability at C1 (100%) indicating that alpha, beta-unsaturated amide moiety is responsible for metabolic lability of ACB (**B**).

The toxicity assessment of ACB was conducted using DEREK software (Leeds, UK) in silico (Figure 3). ACB exhibits structural warnings of skin sensitization (equivocal) due to alpha, beta-unsaturated amide moiety. The previous outcomes indicated that the toxicity (Figure 3A) and the metabolic lability (Figure 3B) are due to the alpha, beta-unsaturated amide moiety that matched. Minor structural changes in the alpha, beta-unsaturated amide moiety or substitution of the group in drug design have the probability to improve the metabolic stability and safety profile of novel derivatives in comparison to ACB (Figure 3).

### 2.3. In Silico ADME Profile

The drug likeliness of ACB was determined by evaluating its ADME properties. Based on the log *p* value as expected by the SwissADME software (http://www.swissadme.ch/; version 1 (accessed on 24 August 2023)), it was determined that the solubility of ACB in water exhibited a low significance level (Log S = −4.69). Furthermore, the anticipated pharmacokinetic parameter for absorption in the gastrointestinal tract is significantly elevated with no permeability across the blood–brain barrier. The bioavailability score is reported to be 0.55. The compound ACB is anticipated to function as an inhibitor for some cytochrome P450 enzymes (namely, CYP2C19, CYP2C9, CYP3A4, and CYP2D6) as well as P-glycoprotein, which is a substrate. It is proposed that ALC is not an inhibitor for the CYP1A2 enzyme. The Log Kp value for skin permeability prediction is −6.98 cm/s. In terms of drug similarity, it adheres to the Lipinski rule. Additionally, it violates the Ghose rule with one violation: MR > 130. Figure S1 displays the ADME radar chart for ACB, while Table S1 provides the corresponding statistics in the Supplementary Materials.

### 2.4. UPLC–MS/MS Methodology Establishment

**Figure 4:**
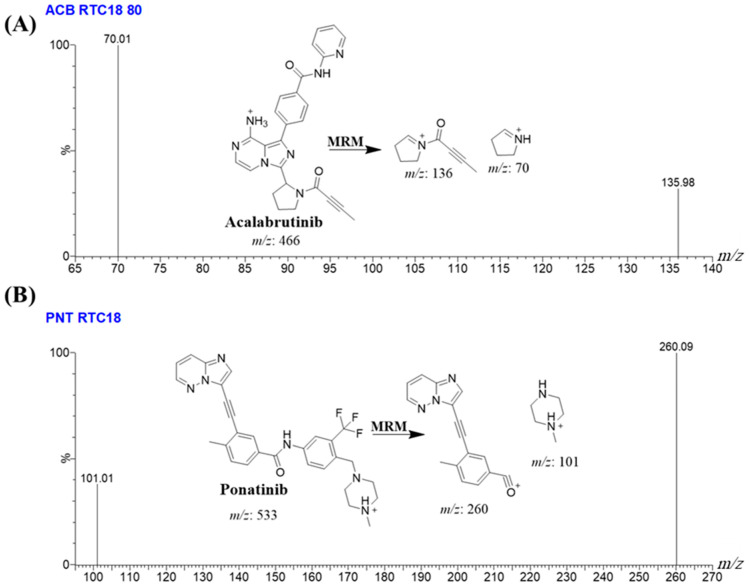
MRM mass spectrum of ACB [M+H]^+^ (**A**) and IS [M+H]^+^ (**B**). The predicted fragmentation behaviors are explained.

The study investigated different stationary phases, encompassing both nonpolar and polar characteristics. These phases included a HILIC DIOL column (2.1 × 100 mm, 1.7 µm) obtained from Fortis™ Technologies Ltd. (Neston, UK) and a polar imidazole column (2.1 × 50 mm, 1.8 µm) acquired from Sepax technologies (Newark, DE, USA). Nevertheless, the polar columns exhibited restrictions in terms of their capability to effectively separate and maintain ACB and IS. Though, the utmost favorable outcomes were obtained using the C18 column, which used a reversed stationary phase. Despite the retention of analytes achieved by employing the C8 column in the UPLC–MS/MS technology for resolving ACB and IS, the targets (ACB and IS) demonstrated inadequate separation of base peaks, extended elution durations, and peak tailing. The ZORBAX RRHD Eclipse Plus C18 (2.1 × 50 mm, 1.8 µm, 95 Å) column, acquired from Agilent Technologies (Santa Clara, CA, USA), demonstrated the most advantageous results in terms of chromatographic peak morphology and elution time. The separation of ACB and IS was achieved in the UPLC–MS/MS approach by employing a chromatographic device with an isocratic mobile phase. The separation was conducted at a flow rate of 0.3 mL/min and a period of 1 min. The calibration graph for ACB exhibited linearity within a concentration range of 1 to 3000 ng/mL. In order to improve the sensitivity of the method of analysis using UPLC–MS/MS, the MRM measurement mode was utilized for the detection as well as quantification of ACB and IS. The aim of this method was to minimize any potential interference caused by matrix components observed in HLMs, as illustrated in Figure 4.

**Figure 5:**
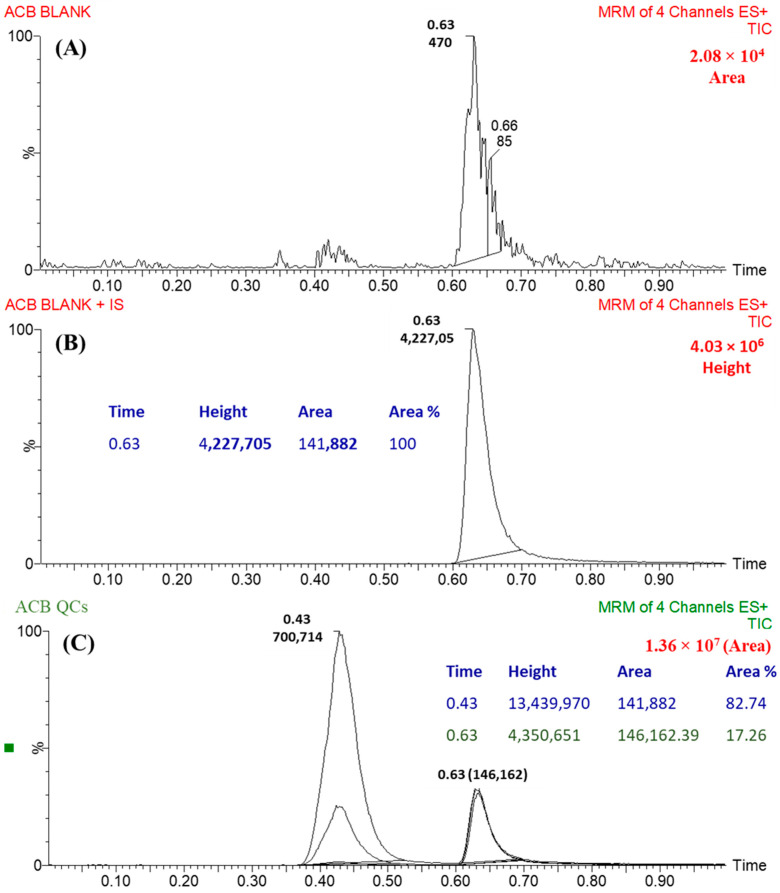
(**A**) The negative control sample entailing of the HLMs matrix did not display any interfering peaks at the elution times of acalabrutinib (ACB) and ponatinib (IS. (**B**) The MRM chromatogram of blank HLMs spiked with IS at 1000 ng/mL. (**C**) The MRM chromatograms of the QCs (3, 900, and 2400 ng/mL) for ACB were superimposed, allowing for the identification of the ACB peaks at 0.43 min. Additionally, the chromatogram displayed a peak corresponding to IS (1000 ng/mL), appearing at 0.63 min.

Ponatinib was utilized as the internal standard (IS) in the estimation of ACB, employing the established analytical UPLC–MS/MS approach for three distinct objectives. It is crucial to emphasize that protein precipitation, as an extraction technique, can be effectively employed for both ACB and IS, resulting in a substantial enhancement in productivity. The yield of ACB was determined to be 100.83 ± 2.66% (RSD: 2.64), while the yield of IS was observed to be 99.17 ± 1.93% (RSD: 3.24%). Furthermore, it was observed that the eluted peaks corresponding to the ACB (0.43 min) and IS (0.63 min) goals were successfully achieved within a time period of less than one minute. The observed result provides empirical support for the efficacy of the UPLC–MS/MS methodology described in this investigation, since it facilitates rapid separation. This methodology facilitates a substantial reduction in the length of the procedure to just one minute, while also utilizing a decreased amount of ACN, hence promoting environmental sustainability. Furthermore, it is crucial to emphasize that the administration of the two analytes, ACB and IS, did not take place concurrently within the same subject. Therefore, the current UPLC–MS/MS technology has the potential to be employed for therapeutic drug monitoring and pharmacokinetic research of ACB. The MRM mass chromatograms of HLMs controls, both negative (Figure 5A) and positive (Figure 5B) for ACB, exhibited a minimal amount of carryover, with peak heights reaching a maximum of 470. Figure 5C displays the MRM mass chromatograms of ACB QSs at 3, 900, and 2400 ng/mL, together with the IS at 1000 ng/mL. The chromatograms have been superimposed in order to facilitate comparison.

### 2.5. Validation Features of the UPLC–MS/MS Approach

#### 2.5.1. Specificity

The efficacy of the UPLC–MS/MS method was verified through the successful differentiation of the analytical peaks of ACB and IS, as depicted in Figure 5. Furthermore, it was found that there was no detectable interference between the analytical peaks of the analytes, namely, ACB and IS, and the matrix components of the HLMs. A slight residual influence resulting from ACB was seen in the negative control MRM mass chromatograms.

#### 2.5.2. Linearity and Sensitivity

**Figure 6:**
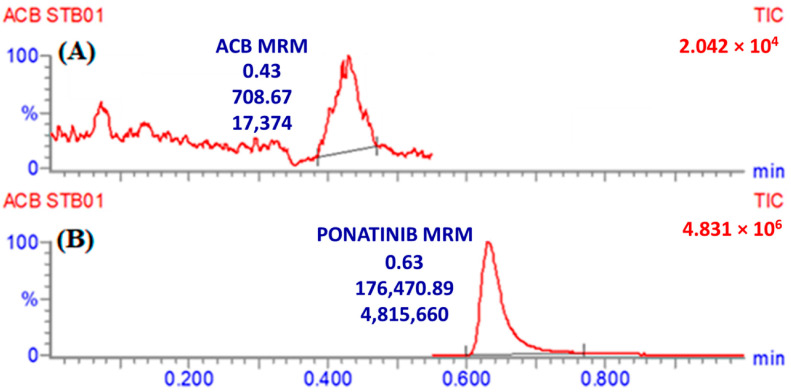
(**A**) The LOQ for ACB standard (1 ng/mL) in the incubation HLMs matrix demonstrates the sensitivity of the UPLC–MS/MS approach currently employed. (**B**) IS chromatographic peak at 1000 ng/mL in HPLC vial while the concentration within the column was estimated to be 5 ng/mL.

The range of the UPLC–MS/MS technique linearity was assessed in a concentration range spanning from 1 to 3000 ng/mL in the incubation HLMs matrix. The achievement of this task involved the introduction of seven ACB CSs into the HLMs matrix, followed by their subsequent analysis as unidentified variables. The obtained linear regression equation may be expressed as y = 0.8692x + 3.441, accompanied by a coefficient of determination (r^2^) value of 0.9989. The creation of the ACB calibration curve involved the utilization of statistical weighting, specifically employing the reciprocal function (1/x), to accommodate the significant variability observed in the CSs. The RSDs of the six repeats (CSs) were determined to be below 7.89%, as described in Table 1. The LOQ and LOD were determined to be 0.97 ng/mL and 0.32 ng/mL, correspondingly, in the HLMs matrix, as seen in Figure 6.

#### 2.5.3. Accuracy and Precision

The assessment of the accuracy and precision of the established UPLC–MS/MS method involved conducting 12 sets, each consisting of four quality control samples, in just one day. Furthermore, a total of six sets, each consisting of four quality control samples, were conducted within a span of three consecutive days. The findings reported were determined to fall within the acceptable range as stated by the validation criteria published by the FDA [24]. The UPLC–MS/MS method developed in this study exhibited inter-day and intra-day accuracy and precision values within the range of −1.00% to 8.36% and −2.87% to 4.11%, correspondingly, as shown in Table 2.

#### 2.5.4. HLMs Matrix Exhibits No Effect on the Extraction and Recovery of ACB in the UPLC–MS/MS Method

The study’s results indicated a significantly higher recovery rate for ACB extraction, with an average score of 100.83% and an SD of 2.66% (RSD less than 2.64%). Similarly, it was noted that the extraction recovery rate of IS exhibited a noteworthy performance, with an average value of 99.17% and an SD of 1.93% (RSD less than 3.67%). HLMs that incorporated the use of ACB and IS exhibited an ME of 100.1 ± 1.75% and 99.63 ± 2.98%, respectively. The normalized ME of the IS was calculated to be 1.01, which falls within the permitted range specified by the criteria established by the FDA. The results of the experiment indicate that the HLMs matrix does not have a substantial influence on the ionization of IS or ACB.

#### 2.5.5. ACB Exhibits Good Stability in the HLMs Matrix and Stock Solution

The assessment of ACB stability in the stock solution, namely, in the DMSO, as well as in the incubation matrix of HLMs, revealed that the optimal stability was attained by preserving the ACB in DMSO at −80 °C for a period of 28 days. The analysis revealed that the RSD% for all ACB samples was shown to be below 3.71% across various storage conditions, as depicted in Table 3. There was no notable reduction observed in the ACB concentration when subjected to various storage conditions, including storage in the autosampler, through three freeze–thaw cycles, short-term storage, and long-term storage. The study findings indicated that the ACB exhibited a significant degree of consistency across time.

### 2.6. The Assessment of the Environmental Sustainability of the Presently Employed UPLC–MS/MS Procedure through the Utilization of the AGREE Program

**Figure 7:**
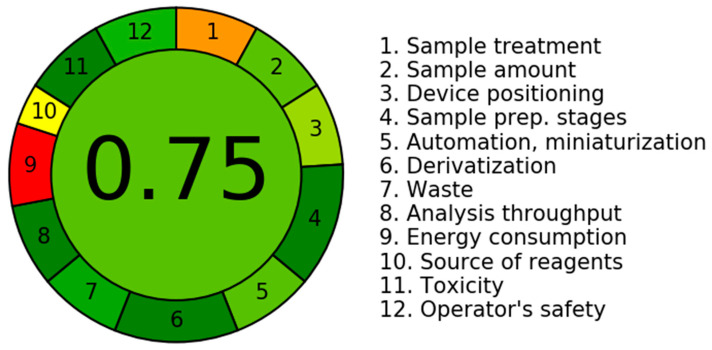
The AGREE program was utilized to showcase the environmentally sustainable characteristics of the established UPLC–MS/MS technology. These colors are representative of the portrayal of twelve separate characteristics.

The evaluation of the sustainability, often known as “greenness,” of the suggested UPLC–MS/MS approach was conducted using the in silico software tool known as AGREE (v.0.5 2020). The program incorporates all twelve requirements specified by the GAC organization (GAC, 10). The software employs a range of numbers ranging from 0.0 to 1.0 in order to allocate values to various aspects of the GAC system. This process facilitates the establishment of analytical metrics that evaluate the extent of environmental sustainability. The outcomes are graphically illustrated by a circular diagram encompassing a diverse spectrum of colors, ranging from red to dark green, symbolizing twelve distinct attributes. Figure 7 depicts the eco-friendly attributes of the UPLC–MS/MS technology presently employed. The recorded ratings for each of the 12 attributes can be seen in Table S2, which is available in the Supplementary Materials. The score of 0.75 was obtained by an evaluation of multiple attributes linked to the current methodology. The score functions as a quantitative metric for assessing the level of environmental sustainability attained through the application of the UPLC–MS/MS technology. A numerical number that tends towards 1.0 indicates a greater degree of environmental friendliness in the analytical procedure. The UPLC–MS/MS technology, which has been recently developed, has a notable degree of ecological sustainability, as indicated by eco-scale scores ranging from 0.75 to 1.00.

### 2.7. In Vitro Metabolic Incubations of ACB with HLMs Matrix

**Figure 8:**
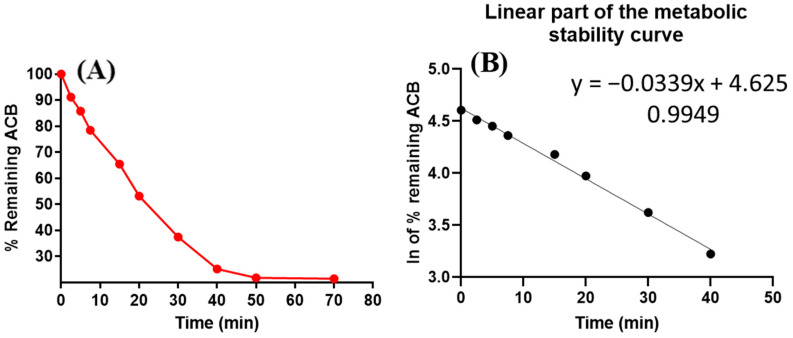
(**A**) The metabolic stability curve of ACB in HLMs matrix. (**B**) The regression equation of the linear segment can be seen using the logarithmic (ln) curve.

The negative control sample exhibited no statistically significant reduction in the concentration of ACB. To assess the metabolic stability of ACB in an in vitro setting, a concentration of 1 μM/mL of ACB was utilized in the in vitro experiments involving human liver microsomes (HLMs). The selection of this particular concentration was made in order to maintain a value lower than the Michaelis–Menten constant. The decision was made with the intention of maintaining a consistent linear relationship between the duration of the in vitro incubation and the rate of ACB metabolism. To mitigate nonspecific protein binding, a concentration of 1 mg/mL of HLMs protein was utilized. Figure 8A depicts the initial metabolic stability curve of ACB. This curve was generated by plotting certain time intervals for quenching, ranging from 0 to 70 min, on the *x*-axis, and the corresponding remaining ratio of ACB on the *y*-axis. The linear section of the previous curve was chosen from the time interval ranging from 0 to 40 min. The aim of this experiment was to create a curve that demonstrates the relationship between the metabolic incubation time points, which ranged from 0 to 40 min, and the natural logarithm of the remaining ratio of ACB. The manifestation of this phenomenon is readily evident in Figure 8B. The outcomes of the study indicated that the slope of the ACB metabolic rate was determined to be 0.0339. The linear equation y = −0.0339x + 4.625 was utilized to illustrate this concept, and it exhibited an r^2^ value of 0.9949, as indicated in Table 4. The calculation of the in vitro t_1/2_ involves dividing the natural logarithm of 2 by the slope. Therefore, the t_1/2_ in an in vitro metabolic incubation was found to be 20.45 min. The calculated ACB (absolute cardiac blood flow) value for Clint proved to be 39.65 mL/min/kg. According to the assessment method devised by McNaney et al. [25], it has been concluded that ACB, a drug with moderate metabolic clearance, is appropriate for the recommended regimen of administering a standard dose of 100 mg twice daily to patients with confirmed B-cell malignancies. Several statistical software programs, including Cloe PK and simulation software, have the capability to predict the in vivo pharmacokinetics of ACB [26].

## 3. Materials and Methods

### 3.1. Materials

Solvents of HPLC purity were used in the UPLC–MS/MS process. The investigation utilized analytical (AR)-grade reference powders, specifically acalabrutinib and ponatinib. Acalabrutinib (ACP-196; 99.88%), the target analyte, and ponatinib (AP24534; 99.43%), the internal standard (IS), were obtained from MedChem, a reliable supplier situated in Princeton, NJ, USA. Sigma-Aldrich, a reliable supplier with headquarters in St. Louis, Missouri, USA, provided the chemicals used in this investigation, which included acetonitrile (ACN), formic acid (HCOOH), ammonium formate (NH_4_COOH), and HLMs. Prior to being used, the HLMs (20 mg/mL) were kept in a refrigeration unit at a constant temperature of −78 °C. Using an internal Milli-Q water purification system from Millipore (Billerica, MA, USA), HPLC-grade water was produced.

### 3.2. UPLC–MS/MS Instrumental Features

The Acquity UPLC system, with a model code UPH and serial number of H10UPH, was employed to attain the chromatographic separation of ACB and IS peaks. The Acquity TQD MS instrument (with model code TQD and serial number QBB1203) was used to perform mass measurement of the target analyte peaks (ACB and IS) after extraction from the incubation HLMs matrix. The UPLC–MS/MS features were tuned to attain efficient separation and good sensitivity of the ACB and IS analytical peaks, as listed in Table 5. The chromatographic properties of the HPLC system were attuned to attain the most favorable separation and sensitivity of the analytical peaks of ACB and IS. These modifications encompassed alterations in the mobile phase composition, pH conditions, and the stationary phase polarity, as outlined in Table 5. The separation of ACB and IS was performed using a ZORBAX RRHD Eclipse Plus C18 column (95 angstroms, 2.1 × 50 mm, 1.8 micrometers) acquired from Agilent Technologies (Santa Clara, CA, USA) with the application of isocratic conditions. The mobile phase A was composed of a solution comprising 15% formic acid (HCOOH) with a concentration of 0.1% in water (H_2_O) and a pH value of 3.2. The mobile phase B consisted of 85% ACN and was administered at a 0.3 mL/min flow rate. The examination of ACB indicated the occurrence of peak tailing and prolonged run time while analyzing a solution containing 10 mM of NH_4_COOH with a pH above 3.2. When the proportion of ACN surpassed 85%, the ACB and IS analytical peaks were indistinguishable. Conversely, a decreased ratio caused a prolonged elution duration.

The mass spectrometry characteristics of the TQD MS were tuned to attain high sensitivity for the detection of ACB and IS analytical peaks. The ESI ionization source was employed in the positive ion mode to enable ion formation, as the chemical structure of ACB and IS contained basic nitrogen atoms capable of capturing protons and forming ions with positive charge. The droplet evaporation in the electrospray ionization (ESI) source was enabled by the use of nitrogen gas, which was formed by a nitrogen generator manufactured by Peak Scientific Company (Scotland, UK). The required vacuum inside the mass analyzer was established by utilizing a vacuum pump supplied by Sogevac, a company based in Murrysville, USA. The UPLC–MS/MS equipment was controlled utilizing the MassLynx package (Version 4.1, SCN 805; Milford, MA, USA) that was utilized as the managing program. The MassLynx software package has two significant software components, namely, IntelliStart^®^ and QuanLynx (Milford, MA, USA). The outputs that were created were analyzed and manipulated using QuanLynx software. The MS calibration of ACB (C_26_H_23_N_7_O_2_) and IS (C_29_H_27_F_3_N_6_O) was attained using IntelliStart^®^ software by employing the direct injection of the WKs of ACB and IS (10 µg/mL) into the mobile phase via combination feature. The mass analyzer detection function of the TQD was employed in multiple reaction monitoring (MRM) to estimate ACB and IS. This was carried out to enhance the selectivity and sensitivity of the UPLC–MS/MS technique that was developed. The analyte ions ACB and IS underwent fragmentation into their particular fragment ions in the collision cell (second quadrupole) of the TQD MS analyzer, utilizing collision gas (argon gas at 99.999% purity). The duration of the mass transition for ACB and IS (parent-to-fragment ions) was 0.025 s. Table 6 shows the MRM features and mass transition properties of ACB and IS.

### 3.3. In Silico Study of ACB Metabolic Lability

The computational assessment of ACB’s metabolic stability was conducted using the P450 software (version 6.6) from Optibrium Ltd. (Cambridge, MA, USA) prior to performing the in vitro metabolic incubation experiment of ACB with HLMs. The significance of executing the in vitro metabolic incubations was confirmed by utilizing the results and data collected from the StarDrop™ software tool. The findings were further refined to determine the composite site lability (CSL), which serves as an indicator of ACB metabolic stability [27]. The use of CSL was employed as a pivotal factor in evaluating the metabolic stability of ACB prior to conducting in vitro metabolic incubation, in order to ascertain the necessity of creating the proposed UPLC–MS/MS method for determining ACB metabolic stability. In order to identify ACB metabolic stability (CSL), the SMILES format of ACB (CC#CC(=O)N1CCC[C@H]1c2nc(c3n2ccnc3N)c4ccc(cc4)C(=O)Nc5ccccn5) was attached to the metabolic software (version 6.6). The metabolic lability at each atom was collected for calculating the CSL [28,29] using the following Equation (1):

(1) CSL=ktotal(ktotal+kw)

where k_w_ is the rate constant for water formation.

The CSL is exhibited in the metabolic landscape and in the P450 column data set. It is an estimate of the ability of the product generation phase in the metabolic cycle of CYP3A4. Thus, a lower CSL value indicates a greater probability of better stability.

### 3.4. In Silico Expectation of the Toxicity of ACB using DEREK Software

The assessment of potential toxicity for ACB was conducted using the DEREK software. Additionally, the software was employed to identify structural warnings associated with ACB, with the aim of suggesting structural alterations that could mitigate the observed toxicity.

### 3.5. In Silico ADME Profile

The SwissADME software (version 1) developed by the Swiss Institute of Bioinformatics in Lausanne, Switzerland, was utilized to propose the Absorption, Distribution, Metabolism, and Excretion (ADME) features of ACB. The software under consideration can be accessed through the web platform located at (http://www.swissadme.ch/ (accessed on 24 August 2023)).

### 3.6. ACB and IS Working Dilutions

At 250 mg/mL (ultrasonication required) and 50 mg/mL (ultrasonication required), respectively, ACB and IS showed their maximal solubility in DMSO. Thus, the stock solutions of IS and ACB, each at 1 mg/mL, were dissolved in DMSO. While the IS solution was created at 10 µg/mL, the ACB working solutions (WKs) were prepared at 1 µg/mL, 10 µg/mL, and 100 µg/mL. The stock solutions of ACB and IS, which were first made at 1 mg/mL, were diluted to create these solutions using the optimized mobile phase. Seven ACB calibration standards (CSs) and four quality controls (QCs) were produced using the WKs.

### 3.7. Establishing of ACB Calibration Standards

Before performing the validation steps for the established UPLC–MS/MS methodology, HLMs were made inactive by being exposed to a 2% concentration of DMSO. The deactivation step was conducted for 5 min at 50 °C. The objective of the deactivation phase was to reduce the potential influence of HLMs on the levels of ACB and IS by inhibiting their metabolic processes [30,31,32]. So as to determine the ACB metabolic stability, a special matrix was developed for HLMs. The procedure consisted of the dilution of 30 µL of HLMs (deactivated) with a metabolic buffer. The metabolic buffer used was composed of 0.1 M of sodium phosphate at pH 7.4, along with 1 mM of NADPH (enzyme cofactor) and 3.3 mM of MgCl_2_. The purpose of this dilution was to recreate the circumstances of the in vitro metabolic incubation of ACB and HLMs.

The ACB calibration standards (CSs) were made by diluting the ACB WKs in a sequential manner using the deactivated HLMs matrix. The aforementioned procedure led to the generation of seven distinct CSs at 1, 15, 100, 200, 500, 1500, and 3000 ng/mL. In addition, four QCs were generated at quantities of 1 ng/mL (representing the lower limit of quantification; LLOQ), 3 ng/mL (representing the lower QC; LQC), 900 ng/mL (representing the medium QC; MQC), and 2400 ng/mL (representing the higher QC; HQC). During the course of this experiment, the concentration of the HLMs matrix was consistently maintained at a level exceeding 90% in order to mitigate the potential effects of matrix dilution that may occur during the in vitro incubation period. QCs were utilized as representative samples with concentrations that were not known. The concentrations of these QCs were estimated by employing the regression equation which was generated from the concurrent injection of ACB CSs. A 100 µL volume of IS WK solution, at 10,000 ng/mL, was added into 1 mL of all ACB QCs and CSs as an IS.

### 3.8. Extraction of ACB and IS from the Incubation HLMs Matrix

ACB and IS were efficaciously separated from the incubation HLMs matrix via the employing of the protein precipitation methodology that involved the application of ACN as a precipitating (proteins) and quenching (metabolic reaction) agent for the HLMs matrix. Consequently, a 2 mL ACN was added to the ACB QCs and CSs. The samples underwent continuous shaking for 5 min to enable the extraction of ACB and IS from the precipitated proteins. Following this, centrifugation was conducted for 12 min using a temperature-regulated centrifuge operating at 4 °C at a rotational speed of 14,000 rpm. The purpose of this centrifugation stage was to facilitate the separation of proteins and achieve clarification of the supernatants. To guarantee the suitability and reliability of the samples for input into the UPLC–MS/MS apparatus, a filtration procedure was performed on all incubates using a syringe filter (0.22 µm pore size). The filtered samples were subsequently transferred into special vials with the aim of injecting them into the UPLC–MS/MS apparatus. Negative control and positive control samples were created utilizing the previously mentioned approach in order to validate that the HLMs matrix constituents do not cause any interference at the elution time of ACB and IS. The experimental sample was designated as the positive control composed of IS within the HLMs matrix. The construction of a calibration curve for ACB involved charting the nominal values of ACB on the *x*-axis and the peak area ratio of ACB to IS on the *y*-axis. The determination of the linearity range of the established ACB CSs was performed by assessing the different validation features and the linear regression equation (y = ax + b; r^2^) of the developed UPLC–MS/MS methodology.

### 3.9. Validation of the Current UPLC–MS/MS Method

The validation of the UPLC–MS/MS technique involved the assessment of its linearity, precision, accuracy, sensitivity, extraction recovery, specificity, stability, and matrix effect. This validation process adhered to the analytical method validation phases as specified in the guidelines provided by the FDA [33,34].

#### 3.9.1. Specificity

The specificity of the present UPLC–MS/MS technology was evaluated by injecting six groups of blank HLM samples following the extraction technique. The purified extracts were loaded into the UPLC–MS/MS apparatus and subjected to analysis with the aim of ascertaining whether any interference was observed with the analytical peaks created by the matrix at the identical retention time as the ACB or IS peaks. Subsequently, the obtained data were juxtaposed with spiked HLM matrix samples that encompassed the desired analytes, namely, ACB and IS. The MRM mode was utilized to address the carryover impact of the targets, ACB and IS, in the MS/MS analyzer. The confirmation of this was achieved through the analysis of the negative control sample HLMs, that were lacking ACB and IS, and subsequent observation of the resultant consequences.

#### 3.9.2. Sensitivity and Linearity

The assessment of linearity and sensitivity of the present UPLC–MS/MS approach involved the generation of 12 calibration curves, utilizing 7 calibration standards, for the analysis of ACB in the HLMs matrix. This assessment was conducted within a single day. Subsequently, the unidentified samples were back-calculated by employing the regression equation derived from the calibration curves. The computing of the LOD and LOQ involved the usage of the slope and the intercept SD of the constructed calibration curve in the HLMs matrix, as outlined in Equations (2) and (3), correspondingly [24].

(2) LOD=3.3×SD of the interceptSlope

(3) LOQ=10×SD of the interceptSlope

The estimation of UPLC–MS/MS method linearity was performed by calculating the coefficient of variation (R^2^) and employing the least-squared method (y = ax + b).

#### 3.9.3. Precision and Accuracy

The assessment of accuracy and precision in the established UPLC–MS/MS technique involved the execution of multiple experiments spanning several consecutive days. In order to examine the precision and accuracy of the ACB QCs, a total of six sets were injected over the course of three consecutive days for inter-day evaluation. Additionally, 12 sets were performed in a single day to measure intra-day accuracy and precision. The assessment of the UPLC–MS/MS method’s accuracy and precision was performed by quantifying them in terms of percentage error (%E) and percentage relative SD (RSD), respectively. The estimated data were obtained through employing Equations (4) and (5), correspondingly.

(4) %Error=(average computed conc.—Expected conc.)Expected conc.×100

(5) %RSD=SDMean

#### 3.9.4. Matrix Effect and Extraction Recovery

The evaluation of the influence of the HLMs matrix on the ionization of ACB and IS was conducted by segregating the samples into two separate cohorts. Group 1′s HLMs matrix was enhanced with the addition of the ACB LQC at 3 ng/mL and IS at 1000 ng/mL. Alternatively, group 2 was created by utilizing the mobile phase in place of the metabolic HLMs matrix. The determination of the normalized matrix effect (ME) of the internal standard (IS) was conducted utilizing Equation (6), whereas the ME for ACB and IS was obtained utilizing Equation (7).

(6) IS normalized ME=ME of ACBME of IS (IS)

(7) ME of ACB or IS=mean peak area ratioGroup 1Group 2×100

The assessment of the efficiency of the ACB extraction method from the HLMs matrix and the influence of HLMs on the extent of ACB ionization were carried out by administering four quality control samples through injection. The efficacy of protein precipitation as the chosen extraction technique for ACB and IS was validated through the execution of six sets of four quality control samples in the HLMs (B), followed by a comparison with four quality control samples obtained in the mobile phase (A). The quantification of the extraction recoveries of ACB and IS was achieved by computing the ratio of B to A, multiplied by 100.

#### 3.9.5. Stability

The running of the current study aimed to assess the stability of ACB in the HLMs matrix by evaluating its concentration at two different quality control levels (LQC and HQC). This analysis was conducted to validate the robustness and dependability of the UPLC–MS/MS methodology under various storage conditions. The estimation was conducted through five repetitions under various laboratory conditions, including storage for both short and long durations, storage in the autosampler, and subjecting the samples to three cycles of freezing and thawing. To assess the freeze–thaw stability, the LQC and HQC samples underwent a series of three freeze–thaw cycles. Every cycle involved subjecting the samples to freezing conditions at −80 °C in a refrigerator, followed by thawing at room temperature prior to further processing. In the context of short-term stability experiments, the LQC and HQC samples underwent processing and analysis subsequent to an 8 h period of storage at ambient temperature on the laboratory benchtop. The assessment of long-term stability involved the placement of the spiked HLMs matrix in a refrigerator set at −80 °C for three months before conducting the analysis. The stability of the prepared samples in the autosampler was determined by subjecting the prepared sample to a storage period of 24 h at 10 °C, followed by loading into the UPLC–MS/MS apparatus.

### 3.10. In Vitro Estimation of the Metabolic Stability of ACB

The determination of Cl_int_ and the in vitro t_1/2_ of ACB was conducted by assessing the remaining ratio of ACB after in vitro metabolism. The accomplishment was realized through the use of an active HLMs incubation matrix comprising NADPH (enzyme cofactor) and MgCl_2_. The experimental protocol for in vitro metabolic incubation consisted of four consecutive steps. During the initial phase of the experiment, a 1 µL volume of ACB was subjected to preincubation with metabolic HLMs matrix. The aforementioned procedure was conducted at a temperature of 37 °C for a duration of 10 min, employing a water bath equipped with a temperature control system. During the initiation stage, a concentration of 1 mM of NADPH was introduced to each individual sample. Subsequently, the samples were transferred to a thermostatic shaking water bath, where they were kept at a constant temperature of 37 °C. During the third step of the experiment, a solution of IS with a volume of 100 µL and a concentration of 1000 ng/mL was introduced into the system prior to the addition of acetonitrile (ACN), which served as the solvent for terminating the reaction. The use of this procedure aimed to achieve a consistent concentration of the IS and minimize the potential impact of metabolic reactions on the IS concentration. In the fourth step, known as termination, a volume of 2 mL of ACN was introduced into all samples at predetermined time intervals. The intervals observed in this study were as follows: 0, 2.5, 7.5, 15, 20, 30, 40, 50, 60, and 70 min. The purpose of this procedure was to inhibit the metabolic reaction and cause the excess proteins to form a solid substance. This step is the first in the extraction process of ACB and IS, as stated in Section 3.6. A negative control was performed by incubating ACB with HLMs and no NADPH, following the protocol previously stated. This was carried out to assess the impact of incubation conditions or matrix components on the concentration of ACB during the in vitro incubation tests.

The equation of the regression line obtained from the concurrent injection of ACB control samples was employed to calculate the residual concentration of ACB. The inclusion of specific time intermissions (ranging from 0 to 70 min) on the *x*-axis constituted a crucial component in the formulation of the ACB stability curve. The *y*-axis of the graph represented the relative concentration of ACB surviving compared to its initial concentration at time zero, which was set as 100%. Subsequently, the segment of the preceding metabolic curve spanning from 0 to 40 min was selected to generate a natural logarithmic curve. A graphical representation was generated to depict the relationship between the natural logarithm (ln) of ACB concentrations and the metabolic incubation time points ranging from 0 to 40 min. By examining the slope of the aforementioned curve, it was possible to approximate the rate constant associated with the metabolic stability of ACB. The in vitro half-life (t_1/2_) was determined using the formula ln2/slope, where the slope obtained from the data was employed for the calculation of the in vitro t_1/2_. The liver tissue mass (26 g) per kg of body weight and the HLMs (45 mg) per gram of liver tissue were incorporated into the calculation of the ACB Cl_int_ (mL/min/kg) [35], as indicated in Equation (8) [25].

(8) $${Cl}_{int,}=0693\;x\frac{1}{t_{½}(min.)}\times \frac{mL\;incubation}{mg\;protein}\times \frac{mg\;microsomal\;proteins}{g\;of\;liver\;weight}\times \frac{g\;liver}{kg\;b.w.}$$

## 4. Conclusions

The current investigation focused on the development and verification of a UPLC–MS/MS technique for measuring ACB concentration in the metabolic HLMs matrix. The aforementioned technique was later utilized to estimate the metabolic stability of ACB. The utilization of UPLC–MS/MS technology exhibited advantageous characteristics such as high sensitivity, specificity, eco-friendliness, and effective retrieval of ACB and IS from the HLMs matrix. Protein precipitation was utilized as the preferable extraction procedure. The use of a low flow rate of 0.3 mL/min, along with a decreased amount of ACN and a significantly shorter elution time of 1 min, has rendered the current UPLC–MS/MS methodology environmentally sustainable. The results derived from the metabolic P450 software were verified by comparing them to the results obtained from in vitro HLMs incubation assays. The findings from the investigation on metabolic stability reveal that ACB possesses a half-life (t_1/2_) of 20.45 min and a moderate clearance (Clint) of 39.65 mL/min/kg. These results suggest that ACB can be categorized as a drug exhibiting a moderate extraction ratio. Consequently, it is assumed that the administration of ACB to patients may not result in the accumulation of doses within the body. Based on the results obtained from computational P450 metabolic and DEREK software analyses, it is recommended that introducing minor structural alterations to the alpha, beta-unsaturated amide moiety or replacing the group during the drug design process could potentially enhance the metabolic stability and safety profile of unique derivatives, in comparison to ACB. Potential future study could be conducted using the current methodology, which involves the use of in vitro metabolic incubations and in silico software tools. These strategies play a crucial role in the progress of innovative pharmaceutical development, specifically in terms of augmenting metabolic stability. The findings derived from the analysis of ACB through in silico software and in vitro incubation studies have proved the effectiveness of utilizing various in silico metabolic software techniques in conserving supplies and reducing labor.

## Figures and Tables

**Table 1:** A concise overview of the back-calculation outcomes for six repeats of acalabrutinib (ACB), denoted as CSs.

ACB (ng/mL)	Mean	SD	RSD (%)	Accuracy (%)	Recovery
1.0	0.99	0.08	7.89	−1.00	99.00
15.0	15.24	0.17	1.12	1.62	101.62
100.0	105.18	2.06	1.96	5.18	105.18
200.0	195.10	1.14	0.59	−2.45	97.55
500.0	515.60	8.83	1.71	3.12	103.12
1500.0	1484.81	11.55	0.78	−1.01	98.99
3000.0	3009.95	19.96	0.66	0.33	100.33
% Recovery					100.83 ± 2.66

**Table 2:** Accuracy and precision of the developed UPLC–MS/MS approach.

ACB (ng/mL)	Intra-Day				Inter-Day			
QCs	1	3	900	2400	1	3	900	2400
Mean	0.97	3.12	899.67	2424.73	0.99	3.19	897.89	2407.37
SD	0.003	0.04	11.18	8.40	0.08	0.05	8.36	32.82
Precision (%RSD)	0.31	1.21	1.24	0.35	7.89	1.44	0.93	1.36
% Accuracy	−2.87	4.11	−0.04	1.03	−1.00	6.33	−0.23	0.31
Recovery (%)	97.13	104.11	99.96	101.03	99.00	106.33	99.77	100.31

**Table 3:** The stability analysis of ACB.

Stability Parameters	3.0	2400.0	3.0	2400.0	3.0	2400.0	3.0	2400.0
	Mean		SD		RSD (%)		Accuracy (%)	
Freeze–thaw Stability	3.06	2382.60	0.09	13.11	2.95	0.55	1.89	−0.72
Autosampler Stability	3.10	2409.77	0.06	21.93	1.80	0.91	3.33	0.41
Long-Term Stability	2.95	2380.45	0.11	9.60	3.71	0.40	−1.56	−0.81
Short-Term Stability	2.97	2384.99	0.10	15.17	3.27	0.64	−0.89	−0.63

**Table 4:** Metabolic stability of acalabrutinib (ACB).

Time Points (Min)	Mean ^a^ (ng/mL)	X ^b^	LN X	Linearity Parameters
0.00	466.85	100.00	4.61	Regression line equation: y = −0.0339x + 4.625
2.50	425.67	91.18	4.51	
5.00	400.46	85.78	4.45	R² = 0.9949
7.50	366.10	78.42	4.36	
15.00	305.41	65.42	4.18	Slope: −0.0339
20.00	248.27	53.18	3.97	
30.00	174.84	37.45	3.62	t_1/2_: 20.45 min and
40.00	117.41	25.15	3.22	Cl_int_: 39.65 mL/min/kg
50.00	101.54	21.75	3.08	
70.00	100.05	21.43	3.06	

^a^ Average of three readings; ^b^ X: Average of the % remaining of ACB in three readings.

**Table 5:** LC–MS/MS tuned parameters.

LC (Acquity UPLC; H10UPH)		MS/MS (Acquity TQD; QBB1203)	
Isocratic mobile phase	0.1% HCOOH in H_2_O (15%; pH: 3.2)	ESI	Positive mode
	85% ACN		Cone gas: 100 L/H flow rate
	Injection volume: 5.0 μL		Capillary voltage: 4 KV
	Flow rate: 0.3 mL/min		RF lens voltage: 0.1 (V)
ZORBAX RRHD Eclipse plus-C18 column	i.d.: 2.1 mm		Extractor voltage: 3.0 (V)
	Length: 50.0 mm		Nitrogen (350 °C) at 100 L/hr
	Particle size: 1.8 μm	Mode	MRM
	T: 22.0 ± 2.0 °C	Collision quadrupole cell	Argon gas at 0.14 mL/min

**Table 6:** MRM mode features for the assessment of ACB and IS.

	Time	Retention Time	MRM Mass Transitions
Mass spectra segment	0.0 to 0.55 min	ACB (0.43 min)	466 → 136 *m*/*z* transition (CE ^a^: 24, CV ^b^: 28)
			466 → 70 *m*/*z* transition (CE: 22, CV: 34)
	0.55 to 1.0 min	IS (0.63 min)	533 → 260 *m*/*z* transition (CE: 20, CV: 34)
			533 → 101 *m*/*z* transition (CE: 30, CV: 34)

^a^ Collision energy (eV), ^b^ Cone voltage (V).

## Data Availability

The manuscript contains all the accessible data.

## Supplementary Materials

The following supporting information can be downloaded at: https://www.mdpi.com/article/10.3390/molecules28207220/s1, Figure S1. The ADME radar chart of ACB obtained from SwissADME software. Table S1. ADME properties of ACB screened by SwissADME software. Table S2. The report sheet for the LC-MS/MS approach has been produced in order to evaluate the environmental sustainability of the method, utilizing individual scores in accordance with the principles outlined in the Green Analytical Chemistry (GAC) recommendations.

## Sample Availability

Samples of the compounds are not available from the authors.
