# Supplementary material for: An Ultrafast UPLC–MS/MS Method for Characterizing the In Vitro Metabolic Stability of Acalabrutinib
Source: Molecules. 2023 Oct 23;28(20):7220. doi: 10.3390/molecules28207220 (PMC10609012; doi:10.3390/molecules28207220)
Supplement: Supplementary file 1 [file molecules-28-07220-s001.zip › molecules-2670009-supplementary.pdf]

Supplementary file

An Ultrafast UPLC–MS/MS Method for Characterizing the In Vitro Metabolic Stability of Acalabrutinib

Mohamed W. Attwa *, Ahmed H. Bakheit, Ali S. Abdelhameed and Adnan A. Kadi

Department of Pharmaceutical Chemistry, College of Pharmacy, King Saud University, P.O. Box 2457, Riyadh 11451, Saudi Arabia

*Correspondence: mzeidan@ksu.edu.sa

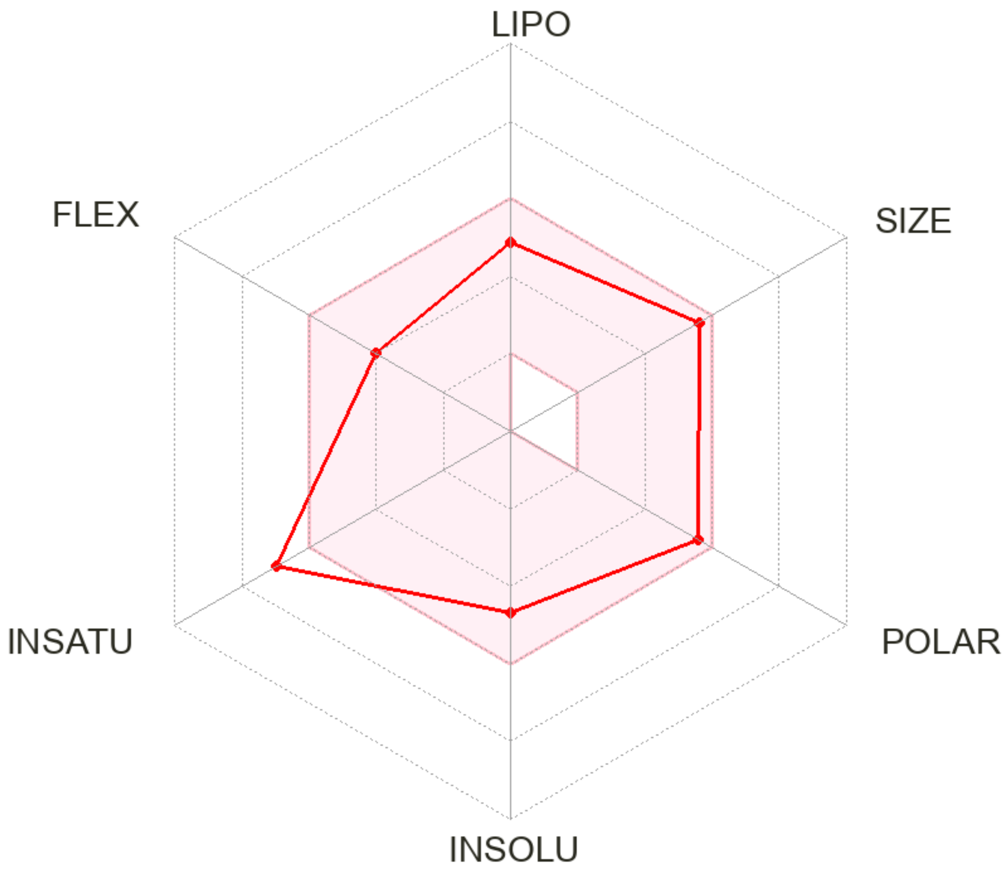

Figure S1. The ADME radar chart of ACB obtained from SwissADME software.

Table S1. ADME properties of ACB screened by SwissADME software.

**Physicochemical Properties**		**Water Solubility**	
**Formula**	$C_{26}H_{23}N_7O_2$	Log S (ESOL)	−4.69
Molecular weight	465.51 g/mol	Solubility	9.52e-03 mg/mL ; 2.04e-05 mol/l
Num. heavy atoms	35	Class	Moderately soluble
Num. arom. heavy atoms	21	Log S (Ali)	−5.19
Fraction Csp3	0.19	Solubility	2.97e-03 mg/mL ; 6.39e-06 mol/l
Num. H-bond donors	2	Class	Moderately soluble
Num. H-bond acceptors	5	Solubility	1.44e-04 mg/mL ; 3.10e-07 mol/l
Num. rotatable bonds	6	Class	Poorly soluble
TPSA	118.51 Å^2	**Medicinal Chemistry**	
Molar Refractivity	136.51	PAINS	0 alert
**Lipophilicity**		Leadlikeness	No; 1 violations: MW>350
Log Po/w (XLOGP3)	3.04	Brenk	1 alert: triple bond
Log Po/w (WLOGP)	2.50	Synthetic accessibility	4.13
Log Po/w (MLOGP)	1.52	**Pharmacokinetics**	
Log Po/w (iLOGP)	3.10	GI absorption	High
Log Po/w (SILICOS-IT)	2.02	BBB permeant	No
Consensus Log Po/w	2.44	P-gp substrate	Yes
**Druglikeness**		CYP1A2 inhibitor	No
Ghose	No; 1 violations: MR>130	CYP2C9 inhibitor	Yes
Lipinski	Yes; 0 violation	CYP2D6 inhibitor	Yes
Egan	Yes	CYP3A4 inhibitor	Yes
Veber	Yes	CYP2C19 inhibitor	Yes
Muegge	Yes	Log Kp (skin permeation)	−6.98 cm/s
Bioavailability Score	0.55		

Table S2. The report sheet for the LC-MS/MS approach has been produced in order to evaluate the environmental sustainability of the method, utilizing individual scores in accordance with the principles outlined in the Green Analytical Chemistry (GAC) recommendations.

Criteria	Score	Weight
1. In order to avoid the necessity of sample treatment, it is recommended to utilize direct analytical procedures.	0.3	2
2. The aims of this study are to attain a limited sample size and a reduced number of samples.	0.75	2
3. Ideally, it is recommended to conduct measurements in their original context, if feasible.	0.66	2
4. The amalgamation of analytical methodologies and operational processes holds promise in terms of energy conservation and reduction in reagent consumption.	1.0	3
5. It is advisable to choose for the utilization of automated and reduced methods.	0.75	2
6. It is recommended to abstain from utilizing derivatization processes.	1.0	3
7. Minimizing the production of a significant volume of analytical waste and implementing efficient management strategies for its disposal are crucial imperatives.	0.88	2
8. Within the domain of analytical chemistry, there exists a predominant inclination towards employing multi-analyte or multi-parameter methodologies, as opposed to those that solely concentrate on a singular analyte.	1.0	2
9. It is imperative to undertake measures aimed at reducing energy use.	0.0	2
10. It is recommended to give priority to the utilization of reagents that are obtained from renewable sources.	0.5	1
11. The elimination or replacement of toxic reagents is crucial.	1.0	2
12. There is a need to enhance the safety measures for operators.	0.8	2
